# Supplementary material for: Learning curve and surgical outcome of robotic assisted colorectal surgery with ERAS program
Source: Sci Rep. 2022 Nov 29;12:20566. doi: 10.1038/s41598-022-24665-w (PMC9709162; doi:10.1038/s41598-022-24665-w)
Supplement: Supplementary file 1 — Supplementary Information 1. [file 41598_2022_24665_MOESM1_ESM.zip › LSC Raw data-Ñ[▒K/108/N_ñw╝╨░O▒Kñσ.pdf]

W86 / W86

臺中榮民總醫院
手術室護理記錄

[redacted]

基本資料

- 室別： 開刀房-21室，手術科別： CRS，手術類別： 常規，日期： 2020/12/09
- 進入時間： 1255 ； 麻醉開始時間： 1300 ； Time Out時間： 1420 ； 劃刀時間： 1421 ； 手術結束時間： 1750 送出時間： 1825 ；
- 麻醉方式： GE

  診斷：UPPER RECTAL CA, 10CM FAV

  手術名稱：Low anterior resection with colo-anal anastomosis Laparoscopy

  手術局部用藥：無

  術前預防性抗生素：
    - 抗生素名稱一： Veterin 1GM IVD由麻醉護理人員執行
    - 時間： 1415
    - 上次簽章: 盧陵怡

  ，追加預防性抗生素：
- 手術醫師： 蔣鋒帆，黃邑霖，楊曙榮，陳凱祥
- 麻醉醫師： VS 劉恆豪
-
    - 刷手護士A ： 羅媗鈴，時間： 1255，接替刷手一： 賴雯惠，時間： 1450，接替刷手二： 紀致均，時間： 1800
    - 流動護士A ： 盧陵怡，時間： 1255，接替流動一： 蘇郁安，時間： 1450，接替流動二： 李苓瑜，時間： 1730
- 更多接替及流動護士： 接替流動三： 蘇郁安，時間： 1800

皮膚狀況

A-V Shunt： 無

術前： 完整

記錄者： 上次簽章: 盧陵怡

術後： 完整

記錄者： 上次簽章: 蘇郁安

手術準備

手術臥式 單一手術臥式

-
    - 手術臥式： 截石臥式
    - 使用附件： 溫毯 38 ℃，頭圈，腳架
    - 皮膚準備： 10%Aq Betadine，Hibiscrub，2% Chlorhexidine
    - 電燒： 使用電燒: 電極片位置： 其它： 導電軟墊，記錄者： 上次簽章: 盧陵怡

止血帶T： 無

植入物

- 植入物名稱/號碼/數量/部位：Ethicon Endo GIA 60-4.1*2/直腸
  Colonscope Long Clip x1/Colon

，記錄者： 上次簽章: 李苓瑜

標本送檢

- 細菌培養 無
- 細胞學 無
- 冰凍切片 無
- 病理標本

  組織名稱一：IMA root

  ，

  組織名稱二：distal cut end

  ，

組織名稱三： rectum

- 截肢 無

標本交付 標本： 1 袋 ， 標本： 0 盒 ， 標本： 2 瓶 ， 細胞學： 0 管 ， Culture Tube： 0 管 ， 被交付醫師： R 陳凱祥，

- 記錄者： 上次簽章: 李苓瑜
- 時間： 1800

傷口術後敷料

傷口敷料： 傷口敷料： 無

- 請於圖上標示部位：

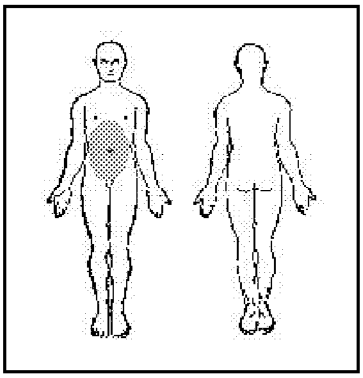

- 傷口敷料： 軟性敷料

導管

導管	天數	導管資訊	插入日期	插入時間	更換日期	拔除日期	拔除時間	拔除原因	預計拔除/更換	預計拔除/更換日期
Epidural PCA(1)	1	部位:硬膜外;	2020/12/09	1310						
Penrose Drain(1)	1	號碼:12 mm.; 部位:骨盆腔;	2020/12/09	1800						
Foley(1)	1	號碼:16 Fr.; 部位:膀胱;	2020/12/09	1800						
A-line (1)	1	號碼:20 G; 部位:左手;	2020/12/09	1315						

用物計數

- 手術中出血量： Minimal c.c.
- 手術中尿量： 1300 c.c.
-
    - 其它： 無

用物計數： 多切口同時縫合

- 1.器械： 正確，2.縫針： 正確，3.刀片： 正確，4.紗布： 正確，5.腹部墊： 未使用，6.紗球： 未使用，7.PEANUTS ： 未使用，8.紗條： 未使用，9.絲帶： 未使用，10.針頭： 未使用，11.Vessel loop： 未使用，12.Boots： 未使用，13.Jelco： 未使用，14.棉條： 未使用，
    - 第一次：
        - 刷手護士 ： 賴雯惠
        - 流動護士： 上次簽章: 李苓瑜
        - 時間： 1725

    ,
    - 第二次：
        - 刷手護士 ： 賴雯惠
        - 流動護士： 上次簽章: 李苓瑜
        - 時間： 1735

    ,
    - 第三次：
        - 刷手護士 ： 賴雯惠
        - 流動護士： 上次簽章: 李苓瑜
        - 時間： 1745

備註

備註： 腹腔鏡NO.2

- 記錄者： 上次簽章: 盧陵怡
- 時間： 1255

填寫護理人員 蘇郁安

正式摘要

	W86 / W86			

doctorRank:B
